# Generalized Gibbs Phase Rule and Multicriticality Applied to Magnetic Systems

**DOI:** 10.3390/e24010063

**Published:** 2021-12-29

**Authors:** Daniele A. Dias, Francisco W. S. Lima, Joao A. Plascak

**Affiliations:** 1 Campus Patos de Minas, Universidade Federal de Uberlândia, Patos de Minas 38700-103, Brazil; daniele@ufu.br; 2 Dietrich Stauffer Computational Physics Lab, Departamento de Física, Universidade Federal do Piauí, Teresina 64049-550, Brazil; fwslima@gmail.com; 3 Departamento de Física, Universidade Federal de Minas Gerais, Belo Horizonte 30123-970, Brazil; 4 Departamento de Física, Centro de Ciências Exatas e da Natureza (CCEN), Universidade Federal da Paraíba, João Pessoa 58051-970, Brazil; 5 Department of Physics and Astronomy, University of Georgia, Athens, GA 30602, USA

**Keywords:** Gibbs phase rule, magnetic systems, phase diagrams

## Abstract

A generalization of the original Gibbs phase rule is proposed in order to study the presence of single phases, multiphase coexistence, and multicritical phenomena in lattice spin magnetic models. The rule is based on counting the thermodynamic number of degrees of freedom, which strongly depends on the external fields needed to break the ground state degeneracy of the model. The phase diagrams of some spin Hamiltonians are analyzed according to this general phase rule, including general spin Ising and Blume–Capel models, as well as *q*-state Potts models. It is shown that by properly taking into account the intensive fields of the model in study, the generalized Gibbs phase rule furnishes a good description of the possible topology of the corresponding phase diagram. Although this scheme is unfortunately not able to locate the phase boundaries, it is quite useful to at least provide a good description regarding the possible presence of critical and multicritical surfaces, as well as isolated multicritical points.

## 1. Introduction

Just a few years after Andrews’ experimental discovery of the critical opalescence in carbon dioxide (which Andrews himself coined as the *critical point*) [1], Gibbs introduced the *phase rule* [2,3]. The so-called Gibbs phase rule (GPR) is solely based on general thermodynamics arguments and can give a remarkable account of the possible number of phases that can coexist for a given system, as well as how many of them can become equal (i.e., critical) at certain conditions. For example, in the temperature–pressure plane, a pure substance (such as water, alcohol, glycerin, carbon dioxide, etc.) can present a line of two coexisting phases, points where three phases coexist, and also points where two phases can become equal. This is in remarkable agreement with the experimental phase diagram of single compounds, where one has lines of gas-liquid, gas-solid, and liquid-solid coexisting phases, only one triple point, and one critical point (a more complete description of such a phase diagram, with water as an example having multiple triple points, will be discussed below in the text).

Despite being almost one and a half centuries old, the GPR is in fact quite useful not only in physics and chemistry, but also in various other areas, including geochemistry [4], geology [5], materials [6], science education [3,7,8,9], and even biological systems [10], just to cite a few examples. Interestingly enough, up to our knowledge, the GPR has been underused in the area of phase transitions and critical phenomena, especially concerning the most symmetric examples of magnetic systems. This lack of interest in the GPR could perhaps be associated with the *apparent* violations of the rule encountered in ferromagnets and even in the helium four-phase diagram [11]. Fisher himself [11] has shown that these violations can be restored by properly taking into account the symmetries involved in the system. However, he left open the question of a practical way to implement these symmetries in the GPR.

It is thus our purpose to derive a generalized phase rule for systems where the usual GPR is violated and apply it to some known spin Hamiltonian models showing that the corresponding phase diagrams are indeed in agreement with this more fundamental thermodynamic behavior. This is achieved by properly taking into account the external fields needed to break the degeneracy of the ground states of the system in order to compute the desired thermodynamic number of degrees of freedom. It is also an interesting feature of the scheme that one can easily identify where the constraints come from. It turns out that such a phase rule could be quite useful in the study of more complex models. In addition, it will also allow the proper description of the phases, the phase coexistence, and the corresponding critical and multicritical behavior present in these systems (which, as we will see below, are sometimes still misnamed and not suitably characterized in the literature).

The structure of this paper is as follows. In the next section, we present a way to introduce the GPR and its modification due to Zernike. In Section 3, we describe a simple scheme to generalize the GPR, which is quite suitable to apply to more symmetric spin Hamiltonians. The corresponding results of some particular models are then discussed in Section 4. Some final remarks are presented in the last section.

## 2. Gibbs Phase Rule

The GPR, especially when no chemical reactions are present, is the subject of many text books (see, for instance, refs. [12,13]) and can be obtained following different approaches [3,7,8,9,14,15]. What one seeks is knowledge of the number of independent thermodynamic variables *f*, or degrees of freedom, which is necessary to completely specify the thermodynamic state of the system. This number is obtained by computing the total number of extensive variables $N_{e}$ minus the number of *constraints* $N_{c}$ eventually imposed on the thermodynamic variables of the system, namely

(1) $$f=N_{e}-N_{c}.$$

For completeness, we will discuss herein one particular approach that seems to be quite suitable to be extended to the magnetic Hamiltonians treated below.

**Figure 1:**
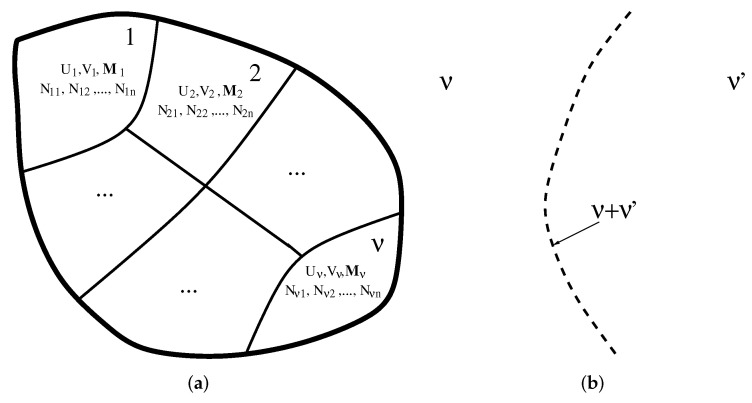
(**a**) A generic system with ν phases. In each phase α=1,2,…,ν there are *n* components with internal energy $U_{\alpha }$, volume $V_{\alpha }$, magnetization $M_{\alpha }$, and number of particles $N_{\alpha i}$ for each component i=1,2,⋯,n. (**b**) Sketch of a hypersurface with dimension $f^{\prime }<f$, where the left has ν coexisting phases and the right has $\nu^{\prime }$ different coexisting phases. Along the dashed line (with dimension $f^{\prime }-1$), $\nu +\nu^{\prime }$ phases coexist.

Consider, initially, a generic system consisting of ν parts or subsystems, in which any part has *n* components, as schematically depicted in Figure 1a. Suppose that all the walls are adiabatic, immovable, impermeable, and perfectly shield any external field. To completely specify the thermodynamic state of this system, one can thus furnish, for each subsystem α, the extensive variables energy $U_{\alpha }$, volume $V_{\alpha }$, magnetization $M_{\alpha }$, and number of particles of the *n* components $N_{\alpha i}$, i=1,2,⋯,n. As in this work we will be concerned with magnetic systems, only the magnetization has been considered; however, if one deals with systems presenting electrical properties, the polarizations $P_{\alpha }$ should also be considered. This means that we have a total of $f=N_{e}=\nu \left(N_{f}+n\right)$ variables, or degrees of freedom, and no constraints at all (note that the constraints we are talking about here refer to relations among the thermodynamic variables and have nothing to do with the walls separating the subsystems). Each subsystem will thus have its own temperature $T_{\alpha }$, pressure $p_{\alpha }$, external field $H_{\alpha }$, and chemical potentials $\mu_{i\alpha }$. Notice that zero external field is compatible with finite magnetizations for ferromagnetic compounds.

It is clear that $N_{f}$ above is also the number of *fields* coming from the corresponding extensive variables $U_{\alpha }$, $V_{\alpha }$, and $M_{\alpha }$. It turns out that $N_{f}$ will in fact determine the dimension of the phase diagram. Although $N_{f}$ is usually larger than three, we can work in some hypersurface projections in this hyperspace of thermodynamic fields. For a simple fluid with no magnetic properties, we have $N_{f}=2$, coming from the temperature and pressure, which are the respective *fields* of the extensive variables $U_{\alpha }$ and $V_{\alpha }$, with a phase diagram in two dimensions. In the most general case, instead of considering the magnetization vector, it is easier to compute $N_{f}$ by counting the number of independent external fields, including those necessary to break the possible degeneracy of the ground state. It should be said that sometimes some of these fields, although conceptually well defined, could have no (or possibly quite difficult) physical realization, as is the case of the helium-4 superfluid related to the phase of the wave function, and the antiferromagnet related to the staggered field.

Suppose now that the internal partitions are made diathermic, movable, and magnetically unshielded. The new equilibrium state will correspond to equal temperatures, pressures, and magnetic fields. In this situation, the number of degrees of freedom reduces to $f=N_{f}+n\nu$, since the fields $N_{f}$ are the same for all subsystems. It is also possible to refine the specification of $N_{f}$ by writing $N_{f}=2+N_{mf}$, with the number 2 coming from temperature and pressure, and $N_{mf}$ giving just the specific magnetic field quantities. However, for practical purposes, it is better to compute $N_{f}$ straight from the beginning, since some models do not have pressure (rigid models), and even temperature (geometric systems), as required variables.

At this point, it is still possible to reduce the number of degrees of freedom by just changing the way of furnishing the component variables. Instead of the extensive number of particle variables $N_{\alpha i}$, we can use the corresponding molar fractions $\eta_{\alpha i}$ (or particle number fraction), where $\sum_{i=1}^{n}\eta_{\alpha i}=1$ for every α phase. This will reduce one thermodynamic variable for each subsystem, resulting in $f=N_{f}+n\nu -\nu$.

Now, considering permeable internal partitions, at equilibrium the subsystems will coexist when all chemical potentials are equal $\mu_{\alpha i}=\mu_{\beta i}$. In this case, we indeed have an actual system with ν coexisting phases, which can be thought of as having no walls at all, as is studied in most thermodynamics text books. This will further decrease the number of degrees of freedom by n(ν−1) and we have finally

(2) $$f=N_{f}+n-\nu .$$

The original GPR for simple fluids is recovered with $N_{f}=2$.

As a simple example, for a single one-component non-magnetic fluid, one has n=1 and the phase diagram has two dimensions for $N_{f}=2$. Thus, the occurrence of one phase has f=2, which corresponds to a surface; the coexistence of two phases has f=1, meaning a line in the phase diagram; and three coexisting phases has f=0, which can occur only in some points on the phase diagram. In addition, when two coexisting phases along a line become identical (by becoming *identical*, we mean here they reach a critical condition or undergo a second-order phase transition), we have an extra constraint and one less degree of freedom (for instance, the densities are equal), implying f=0. As a consequence, we have only critical points and no critical lines. It is quite interesting that the phase diagram of H_2_O with its complex different solid phases presents only two-phase coexisting lines, triple, and critical points [16]. Quadruple points and critical lines are forbidden by Equation (2) and, consequently, do not appear in the phase diagram.

**Figure 2:**
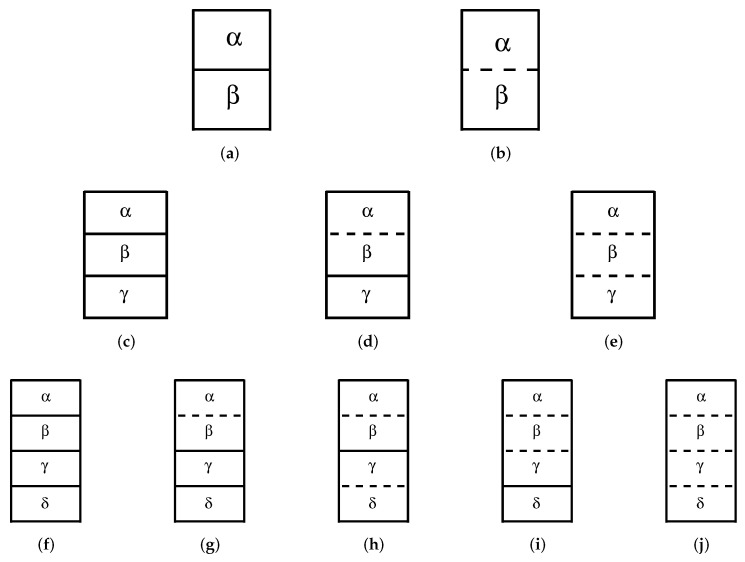
Sketch of two (**upper** row), three (**middle** row), and four (**lower** row) different phases according to the Griffiths scheme [17], where the full lines represent a real interface (a meniscus) between two phases, while the dashed lines represent phases becoming identical, i.e., critical. (**a**) Two coexisting phases; (**b**) two phases becoming identical in a usual critical point; (**c**) three coexisting phases in a triple point; (**d**) one pair of phases becoming identical in a critical end point; (**e**) three phases becoming identical in a tricritical point; (**f**) four coexisting phases in a quadruple point; (**g**) one pair of phases becoming identical resulting, in this case, in a critical end point with 2-phase coexistence [18]; (**h**) two pairs of phases becoming identical resulting in a double critical end point; (**i**) three phases becoming identical forming a tricritical end point; (**j**) four phases becoming identical, which turns out to be a tetracritical point.

Equation (2) can still be extended to more general conditions because depending on the values of the external fields, one or more phases, as shown in Figure 2, can become identical in a critical way. This will imply extra constraints that will further reduce the degrees of freedom. One example has already been discussed above in the case of the critical point of a single component system, and this example is illustrated in Figure 2a,b. Figure 2 also shows some examples of how three and four coexisting phases can become identical, together with the most commonly used nomenclature to designate the corresponding multicritical points. When two phases become identical, as in Figure 2d,g, we have one extra constraint. When three phases become identical, as in Figure 2e,i, we have two extra constraints (if the densities of phases α and β, and phases α and γ are respectively equal, we automatically have the identity of densities β and γ). Four phases becoming identical, as shown in as in Figure 2j, implies three constraints, and so on. This means that Equation (2) reads now

(3) $$f=N_{f}+n-\nu_{1}-\nu_{2}-2\nu_{3}-3\nu_{4}-\cdots \;,$$

where $\nu_{1}$ is the number of single phases, $\nu_{2}$ is the number of two phases becoming identical, $\nu_{3}$ is the number of three phases becoming identical, and so on.

It is worthwhile to mention that in the literature there is not a clear convention on how to name a particular configuration of whole coexisting phases. However, from what has been learned so far, we can say that if we have ν coexisting phases and none of them are critical, this is a *multiple* point, with the Latin terminology for the number of phases (Latin(ν)ple, as in triple, quadruple, quintuple, etc.). If some of the phases become identical, one has a *multicritical* point, with the Greek terminology for the number of equal phases (Greek(ν)critical, as in tricritical, tetracritical, pentacritical, etc.). Figure 2 gives examples for ν=2, ν=3, and ν=4, which makes it easy to generalize for cases of more than four phases (see, for instance, ref. [19] for cases with five and eight phases). In general, if we originally have ν coexisting phases, we can say that: (*i*) if only two phases become identical, one has a critical endpoint with (ν−2)-phase coexistence (ν=3 is a critical end point and ν=2 is a critical point); (*ii*) if only three phases become identical, one has a tricritical endpoint with (ν−3)-phase coexistence (ν=4 is a tricritical end point and ν=3 is a tricritical point); (*iii*) and so on for tetra, penta, etc. critical points; (*iv*) if two different pairs of phases become identical, one has a double critical endpoint with (ν−4)-coexisting phases (ν=4 is a double critical endpoint); (*v*) the same is true for two different sets of three, four, etc. phases becoming identical; (*vi*) if one pair of phases and another different set of three phases become simultaneously identical, we have a critical-tricritical endpoint with (ν−5)-coexisting phases. Generalization for even more complex arrangements is straightforward. The above nomenclature has been thought of considering only *points*, but can be naturally extended to lines, surfaces, volumes, etc. of the respective phase configurations, such as double critical endline, tetracritical endsurface, critical-tricritical endvolume, and so on.

## 3. Generalized Gibbs Phase Rule

The more general GPR given by Equation (3) is known as the Zernike rule [20]. Note that Equation (3) applies for completely non-symmetric systems. However, some symmetry properties among the phases will not reduce the number of degrees of freedom as fast as Equation (3). For example, if, for some reason, phase α is related to phase γ through some special symmetry property and phase β is related to δ by the same symmetry, then when α becomes equal to β, γ automatically becomes equal to δ, and instead of two constraints, only one constraint is involved in the process. As we shall see below, this is what happens in the double critical endpoint of Figure 2f for the Blume–Capel model. This means that the symmetry properties of the Hamiltonians must be carefully taken into account when using the phase rules discussed above.

For the present practical purposes, however, instead of using Equation (3), it is much more convenient to start with Equation (2), where ν phases already coexist, and just count the additional constraints $N_{ad}$ required to accommodate the new specific condition, i.e.,

(4) $$f=N_{f}+n-\nu -N_{ad}.$$

Two different scenarios can now be considered.

(*i*) First, ν phases can coexist in a subset of a hypersurface of dimension $f^{\prime }$ and another different $\nu^{\prime }$ phases coexist in a different subset of the same hypersurface, as schematically shown in Figure 1b. When the proper free energy of any two phases in different subsets are equal, we have one more constraint $N_{ad}=1$, and on the reduced hypersurface $f^{\prime }-1$, one now has $\nu +\nu^{\prime }$ coexisting phases. The special case $\nu =\nu^{\prime }=1$ is equivalent to the usual GPR where two phases coexist on a subspace of a reduced dimension of one unity. By proper free energy, we mean here the corresponding free energy as a function of the suitable fields. That is why we have worked with the chemical potentials in the general case of fluids, because $\mu_{\alpha i}$ are just the molar (or per particle) Gibbs free energy. For more details on what free energy one has to consider in the case of magnetic systems, see Ref. [21].
(*ii*) Second, sometimes the ν (or $\nu^{\prime }$) phases in the hypersubspace can become equal. In this case, $N_{ad}$ should be computed according to the procedure outlined in the previous section, properly taking into account the symmetries of the system.

Actually, it seems that such lack of symmetry analysis has made the use of the GPR rather obscure when treating magnetic models. In addition, the generalized GPR above is more in the direction of a procedure to be followed than a simple application of a closed equation, as in the original GPR. Once the path of the generalized GPR is understood, it turns out to be amazingly simple. Moreover, an interesting point in this method is the ability to identify which thermodynamic quantity the constraints $N_{c}$ and $N_{ad}$ come from.

## 4. Gibbs Phase Rule for Hamiltonian Models

The above procedure is general and can be applied to any system. In this section, we present some theoretical models and discuss the application of the generalized GPR on the corresponding phase diagrams. We will follow the scheme of using Equation (4) and identifying the corresponding constraints in order to compute the desired degrees of freedom *f*. Most of the models have already a consensual phase diagram obtained either from exact results or reliable theoretical approximations, including as computer simulations. However, in some cases there is lack of information regarding the respective phase diagram, and the present generalized GPR can provide some clear insights of what one can expect regarding the type of boundaries of the corresponding phases. We hope that these examples can give a helpful account on how to extend the treatment to either more general or related Hamiltonians.

### 4.1. Ising Model

**Figure 3:**
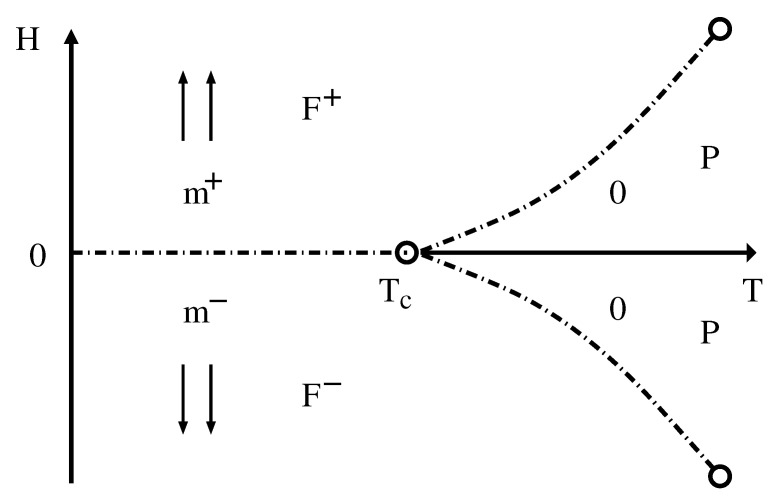
Phase diagram of the Ising model in the temperature *T* vs. field *H* plane. The two ferromagnetic phases (F^+^ and F^−^), with spins aligned along the external field as indicated by the arrows (with magnetizations $m^{+}$ and $m^{-}$), coexist at H=0 along the horizontal dashed-dotted line. The circles are critical points. The other two symmetric dashed-dotted lines are spurious coexistence lines of the ferromagnetic and paramagnetic (P) phases (with null magnetization indicated by the zeros), which are allowed by the GPR (in this case the circle at H=0 would be a triple point instead). The two symmetric lines can eventually terminate at new critical points.

The easiest and mandatory model is the spin-*S* Ising model [22,23,24], which can be written as

(5) $$H=-J\sum_{\langle ij\rangle }\sigma_{i}\sigma_{j}-H\sum_{i=1}^{N}\sigma_{i},$$

where *J* is the exchange interaction, *H* is the external field, and the first sum is over nearest-neighbor sites 〈i,j〉 on a lattice with *N* sites. Each site of the lattice has a spin *S*, taking values $\sigma_{i}=0,\pm 1,\pm 2,\cdots ,\pm S$ for integer *S*, and $\sigma_{i}=\pm 1/2,\pm 3/2,\cdots ,\pm S$ for half-integer *S*. At H=0, the pair interaction term has the time reversal symmetry, so the ground state corresponds to two-fold degenerated ferromagnetic (F^+^ and F^−^) states, where all spins are either aligned in the +S state or the −S state, with magnetization per spin given by $m^{\pm }=\pm S$. The increase of temperature decreases the spin alignment so that $|m^{\pm }|<S$, and the system eventually reaches a paramagnetic (P) phase with no net magnetization $m^{\pm }=0$. The effect of the external field is to break this degeneracy. Thus, besides *H*, one has the temperature *T* as the other thermodynamic field, which comes from the spin–spin interaction part of the energy. Accordingly, the phase diagram has two dimensions ($N_{f}=2$) and Equation (4) reduces to f=3−ν for a one-component system n=1 and no additional constraints $N_{ad}=0$. One phase, which in this case can be either ferromagnetic or paramagnetic, occupies a surface (f=2); two phases coexist along a line (f=1), which can also be seen as reducing f=2 by one constraint that comes from both phases having the same free energy; a critical point, where two phases become identical, can only be located at a point on the coexistence line (f=0), because one has one additional constraint $N_{ad}=1$ coming from the equality of the magnetization (or, equivalently, the order parameter) of both phases. This is a well-known result and is schematically shown in Figure 3.

Note, however, that from the GPR there is also another possible scenario in which the ferromagnetic phases also coexist with the P phase along two symmetric lines (that may also present two symmetric critical points), making the open circle at H=0 a triple point instead. This is not what really occurs in this model, since any non-zero external field completely destroys the phase transition in this system. This is a clear example that the GPR is able to say what is permitted for nature, but not how nature itself decides to behave. Interestingly enough, as we shall see below, the *q*-state Potts model in an external field has a similar behavior as shown in Figure 3 for q>5 with a multiphase coexistence at H=0. However, there is just one critical point for H>0, since there is no symmetry for negative values of the external field, and only a first-order transition line for H<0.

### 4.2. Blume-Capel Model

A more compelling example that quite completely illustrates the use of the generalized GPR in magnetic systems is the Blume–Capel model [25,26]. It is in fact the simplest generalization of the Ising model given by Equation (5), and is obtained by including a crystal field term Δ, so that the Hamiltonian can be written as

(6) $$H=-J\sum_{\langle ij\rangle }\sigma_{i}\sigma_{j}-H\sum_{i=1}^{N}\sigma_{i}+\Delta \sum_{i=1}^{N}\sigma_{i}^{2}.$$

The crystal field is only relevant for S>1/2. If we just consider Equation (2), we have $N_{f}=3$, f=4−ν, and a phase diagram in three dimensions. Thus, a single phase is stable in a volume of the phase diagram (f=3), two phases can coexist on a surface (f=2), three phases coexist along a line (f=1), four phases can coexist only at isolated points (f=0), and there is no possibility of tricritical points (f=−1). This is what we obtain without any symmetry analysis. However, the symmetries of the model play a crucial role in determining the topology of the phase diagram for any value of *S* and some values of *f* above are in fact underestimated (for instance, it is possible to have a line of quadruple points with f=1 and tricritical points with f=0 as well).

First, let us see what the phases of the model are and some exact results one can obtain at zero temperature (for more details see Ref. [27]). For Δ≤0, we have a behavior similar to the Ising model. However, as Δ increases, the spin state σ=0 is more populated for integer *S* and the states σ=±1/2 are more populated for half-integer *S*. Specifically at T=0 and H=0, for $\Delta <\Delta_{0}=dJ$, where *d* is the spatial dimension of the hypercubic lattice, we have a two-fold ground state as in the Ising model. For $\Delta >\Delta_{0}$, all spins are in the σ=0 state for integer *S*, while for half-integer *S*, we have another two-fold ground state with the spins now all aligned either in the σ=1/2 state or σ=−1/2 state. For different values of the external field, one has $\Delta_{0}=dJ+\left|H\right|/S$ for integer *S* and $\Delta_{0}=dJ+\left|H\right|/\left(S+1/2\right)$ for half integer *S*. This clearly means that the phase diagram strongly depends on *S*. Thus, it will be instructive to see in detail the cases S=1 and S=3/2 separately first, and only afterwards generalize to larger spin values.

#### 4.2.1. Spin S=1

**Figure 4:**
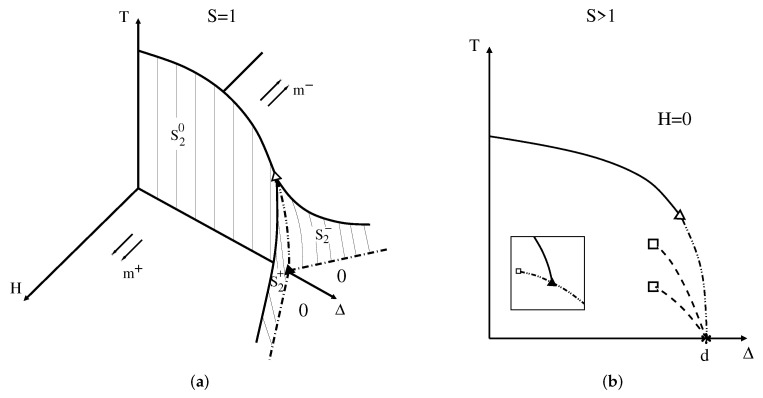
(**a**) Phase diagram of the spin S=1 Blume-Capel model in the crystal field Δ, temperature *T*, and field *H* space. The arrows indicate the two ferromagnetic phases with magnetizations $m^{+}$ and $m^{-}$, and the zeros indicate the paramagnetic phase. The two ferromagnetic phases coexist on the surface $S_{2}^{0}$ at H=0, while the ferromagnetic phases coexist with the paramagnetic phase on the two wings $S_{2}^{+}$ and $S_{2}^{-}$ for positive and negative fields, respectively. Dashed-dotted lines are two-phase coexistence lines that meet at the triple point given by the full triangle located at $\Delta_{0}=dJ$. The dashed-dotted-dotted line is a triple line that ends up at the tricritical point given by the open triangle. Full lines are usual second-order phase transition lines. (**b**) H=0 plane phase diagram for integer spin S>1. There are S−1 dashed lines with four coexisting phases, each of the lines terminating at double critical endpoints (open squares). The star at T=0 and Δ=d is a multiphase point with 2S+1 coexisting phases. The inset shows the forbidden decomposition, according to the generalized GPR, of the tricritical point into a critical endpoint and another double critical endpoint.

The global phase diagram of the spin S=1 Blume–Capel model is schematically shown in Figure 4a. We have three different phases, namely two ferromagnetic phases and one paramagnetic phase, in perfect agreement with the GPR. Single phases occupy volumes in the diagram (f=3); two phases, having the same free energy, coexist on surfaces (f=2), with the two ferromagnetic phases with opposite magnetization (same magnitude) coexisting only at H=0 on $S_{2}^{0}$ surface, and two symmetric wings with the ferromagnetic phases coexisting with the paramagnetic one on $S_{2}^{+}$ and $S_{2}^{-}$ surfaces (the magnetization of the ferromagnetic phases are different from zero, while in the paramagnetic phase no magnetization is present); and three phases coexisting along a line (f=1). Eventually, the magnetizations of the two coexisting phases in each of the three surfaces become equal in critical lines (f=1). Accordingly, the three critical lines meet at the tricritical point (the actual reason Griffiths named this point tricritical). The phases configuration on surfaces $S_{2}^{0}$, $S_{2}^{+}$, and $S_{2}^{-}$ are as in Figure 2a, while at their border critical lines, their configuration is as in Figure 2b. The dashed-dotted-dotted line can be represented by Figure 2c and the open triangle by Figure 2e.

It is interesting to see the role of symmetry regarding this triple line. As discussed above, to derive the Zernike rule (3), the tricritical point should have two constraints in the asymmetric case and therefore seems to violate the GPR for the presence of a tricritical point (f=−1). Here, however, the two ferromagnetic phases always have opposite magnetizations due to the time reversibility of the Hamiltonian at H=0. Thus, if one ferromagnetic phase has zero magnetization, the other phase will automatically also have the same magnetization because of this symmetry, meaning that just one constraint is involved, namely $m^{+}=m^{-}=0$, then reducing f=1 of the triple line to f=0 at the tricritical point.

Another remarkable feature of the generalized GPR is that it does not allow this tricritical point to be decomposed into a critical endpoint and another double critical endpoint, as is shown in the inset of Figure 4b. This new situation would require the impossible constraint $m^{+}=m^{-}\neq 0$.

#### 4.2.2. Spin S=3/2

The main difference now lies in the phases for large values of the crystal field; instead of the paramagnetic phase due to the zero component of the integer spin, there is another ferromagnetic phase coming from the components ±1/2. This means that on the plane H=0 (f=2) and low temperatures, for small (and negative) crystal fields two ferromagnetic phases with opposite magnetizations $m_{1}^{+}$ and $m_{1}^{-}$ coexist, while for large values of crystal fields, two other ferromagnetic phases with smaller magnetization magnitude $m_{2}^{+}$ and $m_{2}^{-}$ coexist. When the free energy of just one of the larger magnetization phases becomes equal to the free energy of any other smaller magnetization phase, as discussed then in the text, the other two phases will also coexist. In this way, only one constraint is necessary to allow for the presence of a line of quadruple points (f=1), which seemed to be forbidden according to the discussion in the beginning of this section.

**Figure 5:**
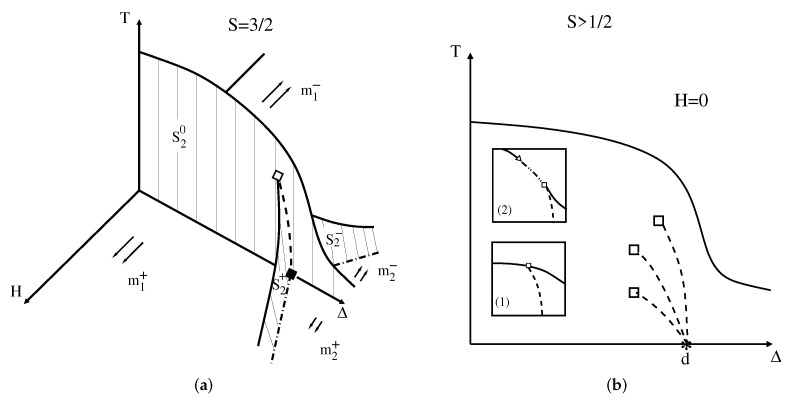
(**a**) Phase diagram of the spin S=3/2 Blume–Capel model in the crystal field Δ, temperature *T*, and field *H* space. The arrows indicate the four ferromagnetic phases with different magnetization magnitudes related by the time reversal symmetry ($m_{1}^{+}$ and $m_{2}^{+}$, and the corresponding inverse ones $m_{1}^{-}$ and $m_{2}^{-}$). For low temperatures, two ferromagnetic phases, with opposite magnetization directions, coexist on the $S_{2}^{0}$ surface for H=0. Along the dashed line on $S_{2}^{0}$ there are four phases coexisting, making it a quadrupole line. The full square at Δ=d is also a quadrupole point, while the open square is double critical endpoint. The two symmetric wings are similar to the wings for the spin S=1 case, the difference now being a coexistence with another ferromagnetic phase instead. The full lines correspond to second-order phase transition lines. (**b**) H=0 plane phase diagram for half-integer spin S>1/2. There are S−1/2 dashed lines with four coexisting phases, each of the lines terminating at double critical endpoints (open squares). The star at T=0 and Δ=d is a multiphase point with 2S+1 coexisting phases. The inset (1) shows a tetracritical point, represented by the open square, and the inset (2) shows a decomposition of the tetracritical point into a critical end point with 2-phase coexistence (open square) and one additional tricritical point (open triangle). Both situations are spurious, but are allowed by the generalized GPR.

A sketch of the phase diagram is shown in Figure 5a. The symmetric wings are surfaces where two ordered phases with different magnetizations coexist. Along the quadruple line at H=0, four phases with magnetizations $m_{1}^{+}$, $m_{2}^{+}$, $m_{1}^{-}$, and $m_{2}^{-}$ coexist, as in Figure 2f. When $m_{1}^{+}=m_{2}^{+}$, due to the time reversal symmetry one also has $m_{1}^{-}=m_{2}^{-}$, so with one additional constraint we obtain f=0, which is in this case a double critical endpoint [28], well illustrated in Figure 2h. Nevertheless, the generalized GPR does not rule out the possibility of a tetracritical point here (Figure 2j is an example), which will be the condition of $m_{1}^{+}=m_{2}^{+}=0$, and the same is true for negative magnetizations, as is schematically shown in the inset (1) of Figure 5b. Therefore, the equality of the magnetizations is one sufficient constraint for multicriticality and we do not expect the specific null value for the magnetizations to be one additional constraint. The decomposition of this tetracritical point into a critical endpoint with 2-phase coexistence (see Figure 2g) and another tricritical point, as depicted in the inset (2) of Figure 5b, is also permitted by the generalized GPR. Although both situations above are spurious and do not happen in the global phase diagram of this system [28,29], such decomposition has actually been seen in the spin-3/2 Baxter–Wu model [19].

#### 4.2.3. *S* > 1 and *S* > 3/2

For larger values of the spin *S*, multiwings will appear in the phase diagram. The projection on the H=0 plane is shown in Figure 4b and Figure 5b for integer and half-integer spins, respectively. S−1 (S−1/2) quadruple lines spring from the multiphase point H=0 and Δ=d and terminate at double critical endpoints [27] for integer (half-integer) *S*.

At the multiphase point H=0, Δ=d, we have 2S+1 coexisting phases, which seems to violate the rule, since we have seen that the maximum number of allowed phases is four. This is in fact an *anomalous* point. At H=0 and Δ=d, if all the spins are aligned in *any* of the states −S,−S+1,⋯,S−1,S, the free energy will be the same. This means that the constraint of equality of the free energy is independent on the value of the spin state. However, outside of this anomalous point, the generalized GPR applies, and as only four phases can coexist, the only possible scenario is the springing of quadruple lines. This is a remarkable theoretical understanding of why the general spin Blume–Capel model presents such behavior at low temperatures.

### 4.3. Two-Dimensional *q*-State Potts Model

The literature is full of systems where the above analysis can be straightforwardly applied. We will briefly discuss below the *q*-state Potts model [30] in order to demonstrate the importance of taking the symmetry breaking fields of the ground states.

The Hamiltonian of the *q*-state Potts model can be written as

(7) $$H=-J\sum_{\langle i,j\rangle }\delta_{\sigma_{i}\sigma_{j}}-H_{1}\sum_{i=1}^{N}\delta_{1,\sigma_{i}},$$

where the first sum is over nearest–neighbor pairs on a square lattice with *N* sites. Each lattice site has a spin variable $\sigma_{i}=1,2,\cdots ,q$, and $\delta_{\sigma_{i}\sigma_{j}}$ is the Kronecker delta, which is 1 when the pair $\sigma_{i}\sigma_{j}$ are in the same state and 0 otherwise. *J* is the ferromagnetic exchange interaction and $H_{1}$ represents the external field applied in the 1 direction. Exact results have shown that at $H_{1}=0$, this model has a second-order phase transition for $q\leq q_{c}$, and a first-order phase transition for $q>q_{c}$, with $q_{c}=4$ [31,32]. The situation, however, for $H_{1}\neq 0$ is not yet completely clear. Due to its complexity, much less work has been devoted to the study of the effects of an applied magnetic field along one of the state components. For example, for $q>q_{c}$ and $H_{1}>0$, a first-order line ending up at a critical point has been theoretically predicted [33] and afterwards detected by Monte Carlo simulations [34]. The use of the generalized GPR could furnish some insights about the possible behavior of the coexisting phases in this model.

At $H_{1}=0$ and below the transition temperature, we have *q* coexisting phases. In order to break the degeneracy of these states, one needs q−1 external fields acting on each state. The reason for having only q−1 instead of *q* fields is because positive fields favor the corresponding state and negative fields tend to suppress this state. Thus, having all q−1 fields with negative values will favor the last state and there is no need for an extra field in this direction. This means that the total number of fields $N_{f}$ (including the temperature), which is also the dimension of the phase diagram, is given by $N_{f}=q$. For a one-component system we now have $f=q+1-\nu -N_{ad}$. One single phase occupies the whole hypervolume of the *q*-dimensional phase diagram, since $N_{ad}=0$ and f=q. On the other hand, having *q* coexisting phases means f=1, which must be a line. This line should be along $H_{1}=0$, as expected.

**Figure 6:**
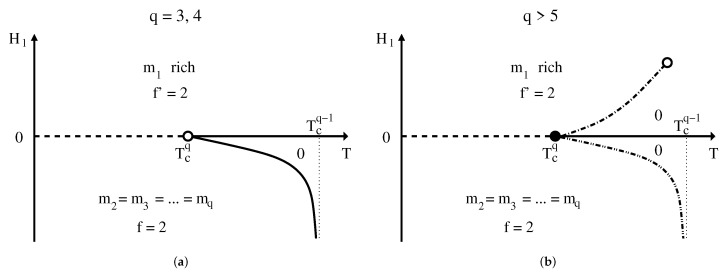
Projection onto the external field $H_{1}$ versus temperature *T* subspace of the phase diagram of the Potts model in two dimensions. Open circles and full lines are second-order phase transitions; at the full circle q+1 phases coexist; along the dashed line and the dashed-dotted-dotted line *q* phases coexist; and along the dashed-dotted line two phases coexist. $m_{i}$ are the magnetizations of the state *i*. For positive fields, the phase is rich in the 1 state alignment, while for negative fields the other states are more populated. *f* and $f^{\prime }$ designate the dimension of the surface. In (**a**), we have q=3,4 and in (**b**), q>5. The phase diagram for q=5 should be similar to that in (**b**) with the dashed-dotted-dotted line becoming a full line.

The effect of the external field is to favor the occupancy of the 1 state for $H_{1}>0$ and to suppress the occupancy of this state for $H_{1}<0$. We can thus expect to have q−1 coexisting phases for negative fields. This means that f=2 on a surface. On this surface, however, we can have, with just one additional constraint, either all magnetizations becoming equal to zero, meaning the existence of a critical line, or the ordered phases coexisting with the paramagnetic one, in another multiple line. It is interesting that in this case, and contrary to the Ising model, depending on the value of *q*, both situations actually occur. Figure 6 sketches a projection of the *q*-dimensional phase diagram of the Potts model in two dimensions on the temperature versus external field plane for q=3,4 in (a) and for q>5 in (b). In (a), positive fields destroy the transition, as in the Ising model, and for negative fields there is a second-order phase transition in the same universality class as the q−1-state model.

For q>5, we have the sketch in Figure 6b, with all lines obeying the generalized GPR. The line and the critical point for positive values of $H_{1}$ has been located for some values of q>4 using the Wang–Landau Monte Carlo method [34]. Nevertheless, the first-order transition line for $H_{1}<0$ still needs to be studied in detail. What we can surely say is that we do not expect any critical points along this first-order transition line, since as $H_{1}\to -\infty$ the model reduces to the q−1-state model, which undergoes another first-order phase transition.

The particular case q=5 is even more interesting, since $H_{1}\to -\infty$ should correspond to a four-state Potts model that has a second-order transition. Thus, the phase diagram should be similar to that shown in Figure 6b, with the dashed-dotted-dotted line becoming now a tetracritical line. The full circle could be a tetracritical endpoint. A less probable scenario, permitted by the generalized GPR, could be a first-order line for H1<0 ending at a tetracritical point only in the limit $H_{1}\to -\infty$. Future works on this model could surely elucidate this still open question.

We end this subsection by saying that the three-dimensional q=3-state and q>3-Potts model should have similar phase diagrams as the q=5-state and q>5-state model, respectively, in two dimensions.

## 5. Final Remarks

It is not surprising that the GPR has been underused in the study of the phase diagrams of magnetic systems. However, the apparent violations of the GPR can be restored by taking into account the proper symmetry-breaking fields of the ground state. Fisher has shown that the inclusion of these fields works nicely for the antiferromagnet, as well as for liquid Helium [11]. In this paper, a generalized GPR, which is an extension of Fisher’s original idea, has been proposed in order to study the possible coexisting phases and the presence of multicriticality in magnetic systems. As examples, we have applied the generalized rule to the Ising, Blume–Capel and Potts models in a detailed way. We believe we have presented the most comprehensive discussion of the complete phase diagram of the general spin Blume–Capel model and the two-dimensional Potts model in an external field. The results for both these models are sparsely discussed in the literature (barely for the Potts model in an external field), with some transition lines still in need of further investigations by more reliable methods.

The Heisenberg model, with three-component spin variables, has a similar phase diagram as the Ising model [35]. However, with Heisenberg spins, we can also consider long-range dipolar interactions *D*. The competition between the short-range exchange interaction *J* and the long-range dipolar interaction *D* will result in a single phase multi-domain configuration, which is actually observed in real magnetic materials. Thus, we have one extra field that, below the critical temperature, will mix the $F^{+}$ and $F^{-}$ phases for H=0, resulting in ferromagnetic phases with null magnetization.

An immediate extension of the Blume–Capel model is the Blume–Emmery–Griffiths model [36,37], which includes a biquadratic term of the form $-K\sum_{\langle ij\rangle }S_{i}^{2}S_{j}^{2}$. The phase diagram is now four dimensional, and three-dimensional projections can be drawn for different values of the biquadratic exchange interaction *K*. In this case, the dimension of the hyperspaces will be increased by one unit, and we will have, for instance, critical, triple, and quadruple surfaces, tricritical lines, and double critical endlines, as well as new sublattice phases populated with zero spin configurations for integer values of *S* [37].

It is clear that the present procedure can be applied to other statistical mechanical models. Among the vast variety of systems, we can cite some with a still increasing experimental attraction: disordered systems such as spin glass [38,39], where the strength of the disorder is treated as an additional field; cold atoms and colloidal systems [40,41], orientational ordering in colloidal molecular crystals and colloidal spin ice [42,43,44,45,46], vortex systems [47,48], old water ice [49], and magnetic ice [50,51].
